# Diet quality in relation to kidney function and its potential interaction with genetic risk of kidney disease among Dutch post-myocardial infarction patients

**DOI:** 10.1007/s00394-024-03355-5

**Published:** 2024-03-02

**Authors:** Anniek C. van Westing, Luc Heerkens, Esther Cruijsen, Trudy Voortman, Johanna M. Geleijnse

**Affiliations:** 1 Division of Human Nutrition and Health, Wageningen University & Research, Wageningen, the Netherlands; 2 Department of Epidemiology, Erasmus MC, University Medical Center Rotterdam, Rotterdam, the Netherlands

**Keywords:** Nutrition, DHD-CVD index, Coronary heart disease, Cohort study, Estimated glomerular filtration rate

## Abstract

**Purpose:**

We examined the relation between diet quality, its components and kidney function decline in post-myocardial infarction (MI) patients, and we explored differences by genetic risk of chronic kidney disease (CKD).

**Methods:**

We analysed 2169 patients from the Alpha Omega Cohort (aged 60–80 years, 81% male). Dietary intake was assessed at baseline (2002–2006) using a validated food-frequency questionnaire and diet quality was defined using the Dutch Healthy Diet Cardiovascular Disease (DHD-CVD) index. We calculated 40-months change in estimated glomerular filtration rate (eGFR, mL/min per 1.73m^2^). We constructed a weighted genetic risk score (GRS) for CKD using 88 single nucleotide polymorphisms previously linked to CKD. Betas with 95%-confidence intervals (CIs) were obtained using multivariable linear regression models for the association between DHD-CVD index and its components and eGFR change, by GRS.

**Results:**

The average DHD-CVD index was 79 (SD 15) points and annual eGFR decline was 1.71 (SD 3.86) mL/min per 1.73 m^2^. The DHD-CVD index was not associated with annual eGFR change (per 1-SD increment in adherence score: -0.09 [95% CI -0.26,0.08]). Results for adherence to guidelines for red meat showed less annual eGFR decline (per 1-SD: 0.21 [0.04,0.38]), whereas more annual eGFR decline was found for legumes and dairy (per 1-SD: -0.20_legumes_ [-0.37,-0.04] and − 0.18_dairy_ [-0.34,-0.01]). Generally similar results were obtained in strata of GRS.

**Conclusion:**

The DHD-CVD index for overall adherence to Dutch dietary guidelines for CVD patients was not associated with kidney function decline after MI, irrespective of genetic CKD risk. The preferred dietary pattern for CKD prevention in CVD patients warrants further research.

**Supplementary Information:**

The online version contains supplementary material available at 10.1007/s00394-024-03355-5.

## Introduction

Chronic kidney disease (CKD) is a growing threat to public health worldwide [1, 2]. Patients with cardiovascular disease (CVD) experience rapid kidney function loss [3, 4], putting them at an increased risk of CKD. In the Alpha Omega Cohort of Dutch post-myocardial infarction (MI) patients, the 6-year risk of premature mortality was 2–3 times higher in patients who experienced moderate kidney function loss, defined as an estimated glomerular filtration rate (eGFR) of 30–59 mL/min per 1.73 m^2^, as compared to patients with a healthy eGFR, defined as an eGFR > 90 mL/min per 1.73 m^2^ [5].

A healthy diet, apart from other lifestyle factors, may be important for CKD prevention in CVD patients. The CORonary Diet Intervention with Olive oil and cardiovascular PREVention (CORDIOPREV) trial focused on the benefits of the Mediterranean diet in patients with stable CVD [6]. During five years of follow-up, a diet rich in extra-virgin olive oil produced less kidney function decline than a low-fat diet that was rich in complex carbohydrates [6]. Long-term follow-up studies of a healthy diet and CKD risk among CVD patients are lacking.

The process of age-related kidney function decline may be accelerated by genetic predisposition, as evidenced by the identification of 308 single nucleotide polymorphisms (SNPs) for CKD in a genome-wide association study (GWAS) [7, 8]. It is unknown to what extent genetic factors could impact the relation of diet with kidney function decline.

We aimed to examine overall diet quality and its components in relation to kidney function decline after MI in Dutch patients of the Alpha Omega Cohort. Diet quality was defined as adherence to the Dutch food-based dietary guidelines [9], which have recently been tailored to CVD patients [10]. We additionally divided the cohort based on genetic CKD risk to investigate the potential impact of genetic predisposition on the associations between diet and kidney function decline.

## Methods

### Study design and study population

The present analysis was performed among patients participating in the Alpha Omega Cohort. This is a prospective cohort study of 4837 Dutch patients (aged 60–80 years old, ~ 80% male) with a history of MI. At baseline (2002–2006), data were collected on demographic factors, lifestyle, medical history, health status, and habitual diet [11]. Blood samples were collected in 2002–2006 (baseline, all patients) and in 2006–2009 (approximately 40 months of follow-up, 60% of the patients, enrolled before August 2005). The Alpha Omega Cohort is conducted according to the principles of the Declaration of Helsinki. All patients provided written and oral informed consent, and the study was approved by the medical ethics committee of the Haga Hospital (The Hague, the Netherlands) and by the ethics committees of participating hospitals.

For the current study, patients were eligible if they had a blood sample at baseline and after 40 months of follow-up (blood samples were collected in the years 2002–2009, *n* = 2488). We then excluded patients without serum cystatin C and/or serum creatinine measurements at baseline and/or at follow-up (*n* = 148). Furthermore, we excluded patients with incomplete dietary data (*n* = 164), and with implausibly high or low energy intakes (< 800 or > 8000 kcal/day for men, < 600 or > 6000 kcal/day for women; *n* = 7). Thus, 2169 patients were left for analyses of the association between the DHD-CVD index and kidney function decline. Supplemental Table 1 shows the characteristics of excluded patients who were not eligible for the present study, mainly because they had no follow-up measurement of kidney function. Additionally, 43 patients had no genetic data, yielding 2126 patients for analyses in subgroups of genetic CKD risk (Supplemental Fig. 1).

### Dietary assessment

Baseline dietary intake was assessed using a validated 203-item semi-quantitative food frequency questionnaire (FFQ) [12]. Food group intake, macronutrients or micronutrients, and energy intake were calculated based on the 2006 Dutch Food Composition Table (NEVO 2006), closest to the time of dietary assessment (2002–2006).

### DHD-CVD index

The Dutch Health Council established dietary guidelines for the general Dutch population in 2015 [9] from which a 15-component adherence score (Dutch Healthy Diet15-index, DHD15-index) was developed by Looman et al. [13]. Recently, the Health Council tailored the dietary guidelines to atherosclerotic CVD patients [10], upon which we modified the DHD15-index to create the Dutch Healthy Diet Cardiovascular Disease index (DHD-CVD index). Compared to the DHD15-index, we changed the score for adherence to the fish guideline because CVD patients are recommended to eat more fish. Furthermore, we added a component for use of cholesterol-lowering plant sterol or stanol-enriched products (any use vs. zero use). An overview of the components of the DHD-CVD index and the scoring system is provided in Supplemental Table 2, and included food items are listed in Supplemental Table 3. For the present analysis, the component “filtered vs. unfiltered coffee” was omitted from the DHD-CVD index because this information was not obtained in the Alpha Omega Cohort. The DHD-CVD index in our analysis has a theoretical range of 0 to 150 points, with higher scores representing better overall adherence to the Dutch guidelines for CVD patients.

### Kidney function assessment

Serum creatinine and serum cystatin C were measured in stored blood samples collected at baseline and after approximately 40 months of follow-up by a central laboratory [14, 15]. Serum cystatin C was measured using a particle-enhanced immunonephelometric assay, and serum creatinine was assessed using the modified kinetic Jaffé method as described in detail elsewhere [5]. GFR was estimated using the 2021 equation of the Chronic Kidney Disease Epidemiology (CKD-EPI) Collaboration which includes both serum creatinine and serum cystatin C [16]. We calculated annual eGFR change for each patient by subtracting baseline eGFR from the eGFR at follow-up and dividing the result by follow-up time in years. Prevalent CKD was defined as eGFR < 60 mL/min per 1.73 m^2^ at baseline.

### Genetic data

Patients were genotyped using the Global Screening Array [17]. Genotype imputation was performed using the 1000 Genomes Project reference panel [18].

We calculated two separate weighted genetic risk scores (GRSs) of CKD based on SNPs that were associated with CKD as reported by a recent GWAS [7]. First, a weighted main GRS was constructed by summing the product of the dosages of the 88 nominally (*p* < 0.05) and genome-wide significant (*p* < 5*10^− 8^) non-ambiguous CKD-related risk alleles and the corresponding log-odds ratios (GRS_all). Second, we calculated a sub-score for genetic risk (GRS_sub), which consists of 16 genome-wide significant CKD SNPs. The selection process of SNPs is depicted in Supplemental Fig. 2, and the SNPs included in the GRSs are listed in Supplemental Table 4. For calculation of the GRSs, we compared the SNP effect alleles of the GWAS [7] with the SNP effect alleles in the Alpha Omega Cohort and harmonised the data accordingly. The effect size belonging to each SNP, as reported by the GWAS, was harmonised in such a way that the interpretation was “higher genetic risk of CKD”. The GRS_all ranged from − 4.161 to 3.950. GRS_all was divided in tertiles (T1: ≤ -0.434; T2: > -0.434 – ≤0.411; T3: >0.411), with T3 representing the group with a high genetic risk of CKD. The GRS_sub ranged from − 3.425 to 3.572. GRS_sub was divided into low and high genetic risk, using the median-split (> -0.00105).

### Assessment of covariates

Data on sociodemographic factors, lifestyle habits, and health status at baseline were collected through self-administered questionnaires as described in detail elsewhere [11]. The highest attained level of education was categorised as elementary, low, intermediate, and high. Smoking status was categorised into never, former, and current. The validated Physical Activity Scale for the Elderly was used to assess physical activity [19], and categorised in three groups: low (< 3 metabolic equivalent tasks [METs]), intermediate (0–5 days/week moderate or vigorous activity [> 3 METs]), and high (≥ 5 days/week moderate or vigorous activity [> 3 METs]). Blood lipids (in mmol/L, i.e., total serum cholesterol, high-density lipoprotein cholesterol [HDL-c], and triglycerides) and plasma glucose (mmol/L) were measured using standard kits (Hitachi 912, Roche Diagnostics, Basel, Switzerland). Low-density lipoprotein cholesterol (LDL-c) was calculated using the Friedewald formula [20]. Patients with a body mass index (BMI) ≥ 30 kg/m^2^ were classified as having obesity. Diabetes mellitus was considered present in case of a self-reported physician’s diagnosis, use of glucose-lowering medication, or elevated plasma glucose (≥ 7.0 mmol/L if fasted > 4 h or ≥ 11.0 mmol/L if not fasted). Blood pressure (mmHg) was measured twice by trained research nurses at the patients’ homes or in the hospital. Systolic blood pressure (SBP) and diastolic blood pressure (DBP) were measured on the left arm with the patient in a seated position using an automated device (Omron HEM-711, Omron Healthcare Europe B.V., Hoofddorp, the Netherlands), and values were averaged. Self-reported medication was coded according to the Anatomical Therapeutic Chemical Classification System (ATC) [21]. Codes for antihypertensive medication comprised C02, C03 (C03C for loop diuretics), C08 and C09 (C09A and B for angiotensin-converting enzyme [ACE] inhibitors and C09C and D for Angiotensin II Receptor Blockers [ARBs]). The code for lipid modifying agents was C10.

### Statistical analysis

We visually checked the distribution of all baseline variables using histograms and QQ-plots. Baseline characteristics and adherence to dietary guidelines are presented for the total analytical sample and across sex-specific tertiles of the DHD-CVD index. Means ± standard deviations (SDs) were used to describe normally distributed data, medians with interquartile ranges (IQR) were used for skewed variables, and n (%) for categorical data.

Beta coefficients with 95% CIs for the association between the DHD-CVD index and kidney function were obtained from multivariable linear regression models. The dependent (outcome) variable in all models was “annual eGFR change”, defined as final eGFR (after 40 months) *minus* baseline eGFR, divided by years of follow-up. Negative betas represent “more annual eGFR decline” and positive betas represent “less annual eGFR decline” with increasing DHD-CVD index.

The DHD-CVD index was analysed per 1-SD increment and in sex-specific tertiles (T1: <77.1; T2: ≥77.1 - <89.2; T3: ≥89.2 for women, and T1: <72.4; T2: ≥72.4 - <84.8; T3: ≥84.8 for men; T1 as reference). We also analysed adherence to guidelines for each individual DHD-CVD component (score) in relation to annual eGFR change, and absolute intake (grams/day) of each DHD-CVD component per 1-SD increment (for vegetables, fruits, whole grains, dairy, fish, tea, liquid fats and oils, and plant sterol or stanol-enriched products) or per 1-SD decrease (for refined grains, solid fats, red and processed meat, sugar-sweetened beverages and fruit juices, alcohol, and sodium intake). Because of low intake, absolute intakes of legumes and nuts was analysed in categories (consumers vs. non-consumers).

For the association of the DHD-CVD index with annual eGFR change, we also used restricted cubic splines (RCS, knots located at 10^th^, 50^th^, and 90^th^ percentile) in men and women separately to assess potential non-linearity. These associations were visualised in graphs. We further studied the distribution of kidney function-related factors and the DHD-CVD index across genetically proxied CKD. We therefore divided GRS_all in tertiles, and used the median-split for GRS_sub. The total DHD-CVD index in relation to kidney function decline was subsequently analysed across categories of genetically proxied CKD risk. Similar analyses were performed for DHD-CVD components among patients at high genetic risk of CKD.

For all analyses, three multivariable models were created. The first two models included potential confounders, which were selected *a priori* based on previous literature and biological knowledge. The basic model (model one) included age, sex, education level (only elementary, low, intermediate, and high), and total energy intake. In model two, we additionally adjusted for smoking status (never, former, current), physical activity (low, intermediate, and high), use of renin-angiotensin aldosterone system (RAAS) drugs (yes, no), and use of lipid-lowering agents (yes, no). In model three, we additionally adjusted for potential intermediates of the DHD-CVD-kidney association: SBP, BMI, diabetes mellitus, and HDL-c. We used model two as the main model. For analyses of individual DHD-CVD components, we additionally adjusted model two for all other DHD-CVD components. In the genetic analyses, we further adjusted model two for the first three genetic principal components.

As additional analyses, the association between the DHD-CVD index and annual eGFR change was repeated in subgroups of patients with and without diabetes, obesity, and CKD. The main analysis was also repeated in a sample without RAAS users and diuretics users because these drugs could improve kidney function and may interact with diet [22–24]. We evaluated the robustness of the associations between DHD-CVD components (score and absolute intake) and annual eGFR change in patients with diabetes, obesity, and CKD.

Missing data of covariables were imputed using multiple imputation with chained equations (with 10 imputations and 10 iterations) using the MICE package [25]. The analyses were performed in each imputed dataset separately, and the estimates were subsequently pooled using Rubin’s rules [26]. We used RStudio version 3.6.0 for all analyses, and a two-sided *p*-value < 0.05 was considered statistically significant.

## Results

### Patient characteristics and habitual food intake

Baseline characteristics of 2169 patients included in the present study are presented in Table 1. The mean age was 68.9 (± 5.4) years, and 80.8% of the patients were male. Compared to patients with the lowest diet quality (T1), patients with the highest diet quality (T3) were more often highly educated, physically active, had lower rates of smoking, and had higher eGFR values. They also suffered less often from diabetes and obesity.

**Table 1:** Baseline characteristics of 2169 patients of the Alpha Omega Cohort and across sex-specific tertiles of the DHD-CVD index

	All patients	DHD-CVD index, score		
		T1 W: <77.1 M: <72.4	T2 W: ≥77.1 - <89.2 M: ≥72.4 - <84.8	T3 W: ≥89.2 M: ≥84.8
	*N* = 2169	*N* = 723	*N* = 723	*N* = 723
**Sociodemographic factors**				
Age, y	68.9 ± 5.40	68.2 ± 5.26	69.0 ± 5.42	69.5 ± 5.46
Women, n(%)	417 (19.2)	139 (19.2)	139 (19.2)	139 (19.2)
Education^a^, n(%)				
Only elementary	446 (20.7)	171 (23.8)	147 (20.5)	128 (17.7)
Low	779 (36.1)	251 (34.9)	288 (40.2)	240 (33.2)
Intermediate	671 (31.1)	221 (30.7)	213 (29.7)	237 (32.8)
High	263 (12.2)	77 (10.7)	69 (9.6)	117 (16.2)
**Lifestyle**				
Smoking status, n(%)				
Never	360 (16.6)	90 (12.4)	120 (16.6)	150 (20.7)
Former	1481 (68.3)	466 (64.5)	514 (71.1)	501 (69.3)
Current	328 (15.1)	167 (23.1)	89 (12.3)	72 (10.0)
Physical activity^a^, n(%)				
Low	856 (39.6)	322 (44.6)	283 (39.3)	251 (35.0)
Intermediate	807 (37.4)	269 (37.3)	278 (38.6)	260 (36.2)
High	497 (23.0)	131 (18.1)	159 (22.1)	207 (28.8)
**Blood lipids**^**a**^, **mmol/L**				
Total serum cholesterol	4.75 [4.19, 5.33]	4.77 [4.22, 5.36]	4.71 [4.16, 5.34]	4.77 [4.18, 5.29]
LDL-cholesterol	2.64 [2.17, 3.17]	2.67 [2.16, 3.20]	2.64 [2.18, 3.15]	2.62 [2.16, 3.16]
HDL-cholesterol	1.21 [1.03, 1.43]	1.21 [1.04, 1.44]	1.19 [1.03, 1.43]	1.21 [1.03, 1.42]
Triglycerides	1.63 [1.21, 2.26]	1.60 [1.25, 2.29]	1.65 [1.21, 2.23]	1.67 [1.18, 2.29]
**Other cardiovascular factors**				
SBP^a^, mmHg	143 ± 21.2	143 ± 21.2	144 ± 21.7	144 ± 20.5
DBP^a^, mmHg	81.5 ± 10.7	81.6 ± 10.3	81.4 ± 11.1	81.5 ± 10.6
BMI^a^, kg/m^2^	27.6 ± 3.61	27.8 ± 3.76	27.7 ± 3.72	27.4 ± 3.33
Obesity^a,b^, n(%)	483 (22.3)	175 (24.2)	165 (22.8)	143 (19.8)
Plasma glucose^a^, mmol/L	5.46 [4.96, 6.35]	5.55 [5.01, 6.50]	5.41 [4.92, 6.25]	5.42 [4.95, 6.28]
Diabetes mellitus^c^, n(%)	394 (18.2)	134 (18.5)	136 (18.8)	124 (17.2)
**Kidney function**				
eGFR, mL/min per 1.73 m^2^	87.0 [71.4, 99.5]	86.0 [71.0, 99.2]	87.6 [72.4, 99.3]	87.6 [70.3, 100.0]
Serum creatinine, µmol/L	84.0 [72.0, 101.0]	86.0 [73.0, 102.0]	84.0 [71.0, 100.0]	82.0 [71.0, 100.5]
Serum cystatin C, mg/L	0.92 [0.82, 1.10]	0.93 [0.82, 1.10]	0.91 [0.82, 1.00]	0.92 [0.82, 1.10]
**Medication use, n(%)**				
Antihypertensives	1887 (87.0)	640 (88.5)	627 (86.7)	620 (85.8)
ACE-inhibitors	918 (42.3)	325 (45.0)	300 (41.5)	293 (40.5)
ARBs	287 (13.2)	79 (10.9)	109 (15.1)	99 (13.7)
Diuretics	442 (20.4)	150 (20.7)	140 (19.4)	152 (21.0)
Lipid-lowering agents	1872 (86.3)	621 (85.9)	630 (87.1)	621 (85.9)

Values are means ± SDs for normally distributed variables, medians [IQRs] for skewed variables, or n (%) for categorical variables. ^a^ Part of the cohort had missing values for education (*n* = 10), physical activity (*n* = 9), total serum cholesterol (*n* = 11), LDL-c (*n* = 108), HDL-c (*n* = 11), triglycerides (*n* = 11), SBP (*n* = 3), DBP (*n* = 3), BMI and obesity (*n* = 2), plasma glucose (*n* = 17). ^b^ Obesity is defined as BMI ≥ 30 kg/m^2^. ^c^ Diabetes mellitus is defined as a self-reported physician’s diagnosis, use of glucose-lowering medication or elevated plasma glucose (≥ 7.0 mmol/L if fasted > 4 h or ≥ 11.0 mmol/L if not fasted). Abbreviations: DHD-CVD, Dutch Healthy Diet for cardiovascular disease patients; LDL-c, low-density lipoprotein cholesterol; HDL-c, high-density lipoprotein cholesterol; SBP, systolic blood pressure; DBP, diastolic blood pressure; BMI, body mass index; eGFR, estimated glomerular filtration rate; ACE-inhibitors, angiotensin-converting enzyme inhibitors; ARBs, angiotensin II receptor blockers

Adherence to individual dietary guidelines (scores) and absolute intakes (grams/day) of foods and drinks in 2169 patients with a history of MI and across sex-specific tertiles of the DHD-CVD index are presented in Table 2. Patients scored on average 79 ± 15 points on the DHD-CVD index out of a maximum score of 150. On average, patients adhered best to guidelines for limiting red meat and alcohol intake (median scores of 10 out of 10 points), and least to guidelines for sufficient legumes and nuts intake (median scores < 2.5 points).

**Table 2:** Adherence to individual dietary guidelines (scores) and absolute intakes (grams/day) of foods and drinks in 2169 patients of the Alpha Omega Cohort and across sex-specific tertiles of the DHD-CVD index

	All patients	DHD-CVD index^a^		
		T1 W: <77.1 M: <72.4	T2 W: ≥77.1 - <89.2 M: ≥72.4 - <84.8	T3 W: ≥89.2 M: ≥84.8
	*N* = 2169	*N* = 723	*N* = 723	*N* = 723
Total DHD-CVD score	79.4 ± 14.6	63.4 ± 7.91	79.6 ± 3.93	95.2 ± 7.57
**Adherence to individual dietary guidelines**				
Vegetables ≥ 200 g/d, score	4.49 ± 1.95	4.11 ± 1.82	4.47 ± 1.91	4.88 ± 2.05
Fruit ≥ 200 g/d, score	5.50 [2.14, 10.0]	3.52 [0.83, 6.03]	5.48 [2.51, 10.0]	8.24 [4.97, 10.0]
Grain products, score No consumption of refined cereal products OR Ratio of whole grains to refined grains ≥ 11	6.65 [5.52, 9.14]	6.26 [4.78, 9.01]	6.58 [5.55, 8.83]	7.22 [5.81, 9.37]
Legumes ≥ 10 g/d, score	2.18 [0.00, 6.26]	0.00 [0.00, 4.38]	1.27 [0.00, 5.79]	4.40 [0.00, 7.94]
Unsalted nuts ≥ 15 g/d, score	1.18 [0.00, 1.84]	0.52 [0.00, 1.84]	1.18 [0.00, 1.84]	1.18 [0.52, 4.72]
Dairy 300–450 g/d, score	7.18 [5.00, 10.0]	6.27 [3.54, 9.08]	7.26 [5.20, 10.0]	7.90 [5.87, 10.0]
Fish ≥ 21 g/d, score	5.31 [2.07, 8.09]	4.46 [0.99, 7.26]	5.19 [1.88, 7.70]	7.26 [3.64, 10.0]
Black or green tea ≥ 450 g/d, score	3.33 [0.39, 10.0]	1.47 [0.00, 4.17]	3.33 [0.47, 10.0]	8.01 [2.51, 10.0]
Fats and oils, score No consumption of butter, hard margarines and cooking fats OR Ratio of liquid cooking fats to solid cooking fats ≥ 13	1.45 [0.15, 10.0]	0.36 [0.00, 1.69]	1.34 [0.24, 10.0]	10.0 [1.26, 10.0]
Red meat ≤ 45 g/d, score	10.0 [8.66, 10.0]	10.0 [7.66, 10.0]	10.0 [9.04, 10.0]	10.0 [9.60, 10.0]
Processed meat 0 g/d, score	5.55 [0.97, 7.34]	3.32 [0.00, 6.23]	5.60 [1.30, 7.19]	6.37 [4.59, 8.22]
Sugar-sweetened beverages and fruit juices 0 g/d, score	3.58 [0.00, 6.87]	2.60 [0.00, 6.09]	3.47 [0.00, 6.78]	4.12 [0.88, 7.36]
Alcohol ≤ 10 g/d, score	10.0 [5.84, 10.0]	8.99 [0.00, 10.0]	10.0 [7.16, 10.0]	10.0 [8.28, 10.0]
Sodium ≤ 1.9 g/d, score	8.68 [6.15, 10.0]	7.56 [4.97, 10.0]	8.89 [6.60, 10.0]	9.30 [7.03, 10.0]
Plant sterol or stanol-enriched products, n(%) with 10 points	877 (40.4)	131 (18.1)	276 (38.2)	470 (65.0)
**Absolute intake of DHD-CVD components**				
Vegetables, g/d	85.3 [63.7, 111.5]	79.2 [54.5, 104.1]	84.5 [65.1, 110.4]	94.8 [72.0, 118.1]
Fruits, g/d	110.0 [42.8, 247.1]	70.3 [16.6, 120.5]	109.5 [50.3, 242.5]	164.9 [99.4, 289.8]
Whole grains^b^, g/d	119.8 [88.0, 160.6]	107.4 [77.6, 159.4]	120.1 [88.3, 160.5]	127.1 [89.5, 162.3]
Refined grains, g/d	29.1 [15.2, 53.3]	34.9 [15.7, 63.4]	30.9 [16.1, 54.5]	25.0 [13.6, 44.0]
Legumes consumers, n(%)	1135 (52.3)	293 (40.5)	364 (50.3)	478 (66.1)
Intake among consumers, g/d	6.2 [4.1, 8.7]	5.1 [3.4, 7.4]	5.8 [4.2, 8.7]	6.8 [4.5, 9.4]
Nuts consumers, n(%)	1507 (69.5)	439 (60.7)	506 (70.0)	562 (77.7)
Intake among consumers, g/d	2.6 [1.8, 7.1]	1.8 [0.8, 2.8]	2.5 [1.8, 3.5]	2.8 [1.8, 7.1]
Dairy, g/d	301 [193, 421]	280 [165, 450]	292 [198, 413]	324 [217, 412]
Fish, g/d	11.1 [4.4, 16.7]	9.4 [2.1, 15.2]	10.9 [3.9, 16.2]	15.2 [7.7, 24.2]
Fatty fish, g/d	6.8 ± 9.2	5.3 ± 7.9	6.4 ± 8.4	8.7 ± 10.6
Lean fish, g/d	7.7 ± 8.8	5.8 ± 6.8	7.4 ± 9.1	10.0 ± 9.9
Tea, g/d	150.0 [17.5, 450.0]	66.1 [0.0, 187.5]	150.0 [21.0, 450.0]	361 [113, 450]
Liquid fats, g/d	21.7 [13.5, 33.5]	18.6 [9.2, 30.1]	22.1 [14.1, 32.9]	25.4 [16.0, 37.3]
Solid fats, g/d	8.61 [0.60, 22.1]	17.5 [7.5, 31.7]	9.18 [1.12, 21.2]	1.11 [0.00, 9.82]
Red meat, g/d	37.1 [20.0, 52.4]	42.1 [22.7, 57.9]	37.3 [20.2, 50.3]	31.3 [16.4, 47.2]
Processed meat, g/d	22.3 [13.3, 45.2]	33.4 [18.8, 51.5]	22.0 [14.0, 43.5]	18.2 [8.90, 27.1]
Sugar-sweetened beverages and fruit juices, g/d	160.5 [78.3, 273.5]	185.0 [97.7, 320.7]	163.2 [80.4, 276.9]	146.9 [66.0, 228.0]
Alcohol, g/d	7.9 [1.5, 18.1]	11.4 [1.8, 31.0]	6.9 [1.2, 15.4]	6.5 [1.3, 13.1]
Sodium, mg/d	2222 (659)	2373 (720)	2187 (640)	2107 (583)
Plant sterols/stanol product consumers, g/d	0.00 [0.00, 13.50]	0.00 [0.00, 0.00]	0.00 [0.00, 13.24]	7.50 [0.00, 20.52]
**Other**				
Energy intake, kcal/day	1875 [1566, 2233]	1974 [1624, 2390]	1835 [1556, 2209]	1825 [1522, 2120]
Protein, g/d	69.2 [58.1, 81.6]	70.4 [58.1, 86.0]	68.3 [57.9, 80.6]	68.1 [58.2, 80.2]
Phosphorus, mg/d	1305 [1088, 1568]	1303 [1059, 1628]	1302 [1075, 1551]	1310 [1121, 1538]
Potassium, mg/d	3194 [2662, 3758]	3130 [2559, 3711]	3152 [2651, 3755]	3288 [2788, 3826]

Values are means ± SDs for normally distributed variables, medians [IQRs] for skewed variables, or n (%) for categorical variables. ^a^ The total DHD-CVD score in this project does not include coffee. ^b^ Whole grains also partly included refined grain products, such as brown bread and multigrain bread. Abbreviations: DHD-CVD, Dutch Healthy Diet for cardiovascular disease patients

### DHD-CVD index, its components and kidney function decline

**Fig. 1:**
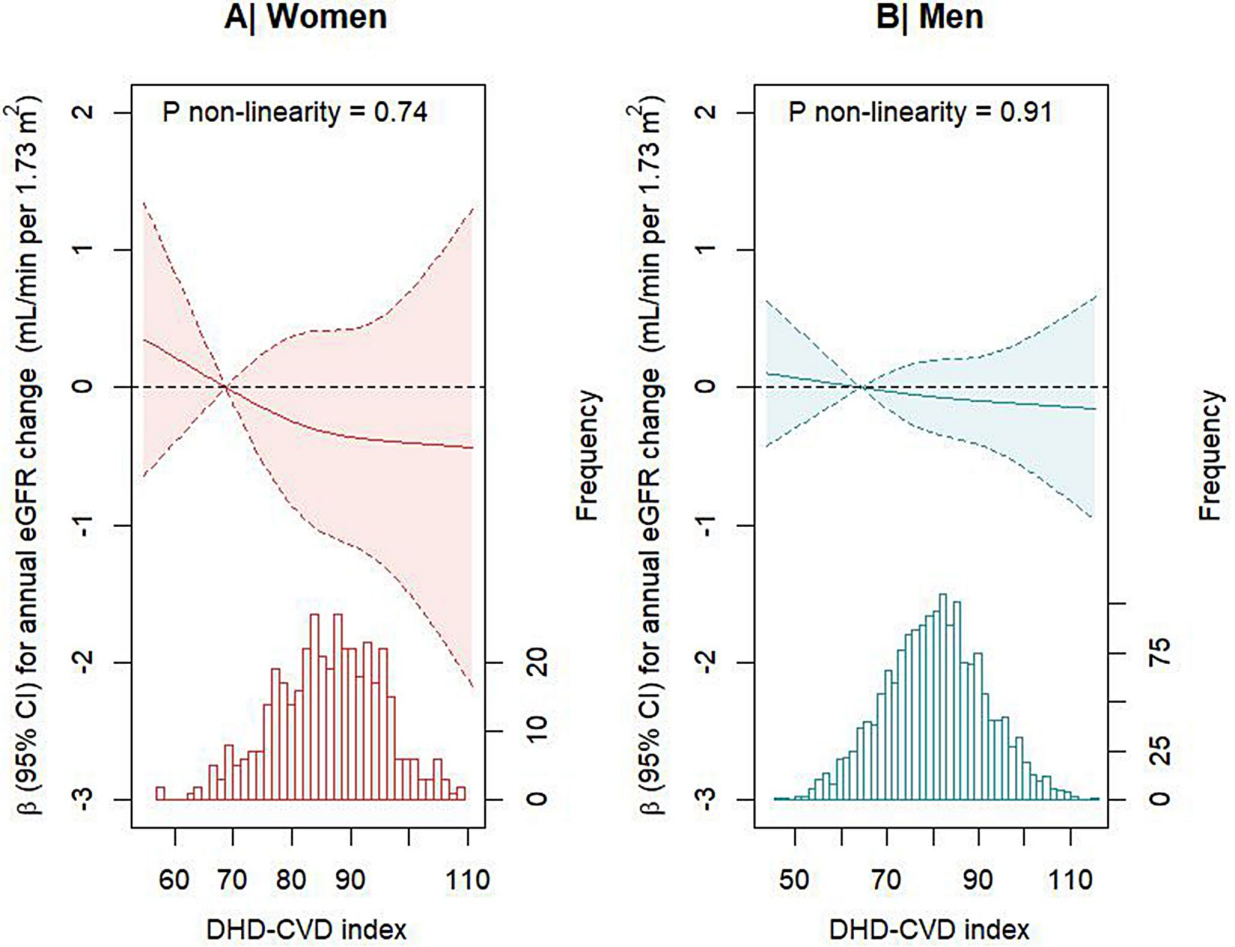
Continuous associations of the DHD-CVD index with differences in annual eGFR change in female (*n* = 417, panel **A**) and male (*n* = 1752, panel **B**) patients of the Alpha Omega Cohort. Solid lines represent beta coefficients and dashed lines represent 95% CIs. The histogram represents the distribution of the DHD-CVD score. Three-knot restricted cubic splines was used, with the median of tertile 1 (69 for women and 64 for men) as reference point. Betas were adjusted for age, education, energy intake, smoking status, physical activity, lipid-lowering medication use, and renin-angiotensin-aldosterone blockers. Abbreviations: eGFR, estimated glomerular filtration rate; CI, confidence interval; DHD-CVD index, Dutch Healthy Diet for cardiovascular disease patients

Patients in the top sex-specific tertile of the DHD-CVD index score had slightly more kidney function decline than patients in the bottom tertile. After multivariable adjustment, this difference was not statistically significant (model 2; beta_T3 vs. T1_ -0.08 [95% CI -0.49;0.33], Table 3; Fig. 1).

**Table 3:** The association between the DHD-CVD index per 1-SD increment in adherence score and in sex-specific tertiles and differences in annual eGFR change in 2169 patients of the Alpha Omega Cohort

	Per 1-SD^a^ increment in adherence score	DHD-CVD index			*P*_trend_
		T1	T2	T3	
Mean ± SD annual eGFR change	-1.71 ± 3.86	-1.71 ± 4.03	-1.57 ± 3.73	-1.85 ± 3.81	
Model 1^b^	-0.05 (-0.22,0.12)	Ref	0.23 (-0.17,0.63)^e^	-0.03 (-0.43,0.38)	0.89
Model 2^c^	-0.09 (-0.26,0.08)	Ref	0.20 (-0.20,0.61)	-0.08 (-0.49,0.33)	0.71
Model 3^d^	-0.08 (-0.25,0.09)	Ref	0.22 (-0.18,0.62)	-0.07 (-0.48,0.34)	0.73

^a^ 1-SD equals 15 points. ^b^ Adjusted for age, sex (2 categories), education (3 categories), and energy intake. ^C^ Model 1 plus additionally adjusted for smoking status (3 categories), physical activity (3 categories), lipid-lowering medication use (2 categories), and renin-angiotensin-aldosterone system blockers (2 categories). ^d^ Model 2 plus additionally adjusted for systolic blood pressure, body mass index, diabetes (2 categories), high-density lipoprotein cholesterol. ^e^ Beta coefficient (95% confidence interval) obtained from linear regression models (all such values). Abbreviations: DHD-CVD index, Dutch Healthy Diet for cardiovascular disease patients; SD, standard deviation; eGFR, estimated glomerular filtration rate

Table 4 shows results for adherence to the individual components of the DHD-CVD index. Patients who adhered to the guideline for nut consumption had less annual decline in kidney function, with a protective association (beta) of 0.17 mL/min per 1.73m^2^ (95%CI -0.004, 0.34) per 1-SD in adherence score. Adherence to the guideline for reducing red meat consumption also showed a protective association (beta of 0.21 [0.04, 0.38]), which was statistically significant. However, more kidney function decline was observed in patients who adhered to guidelines for legumes (-0.20 [-0.37,-0.04]) and dairy (-0.18 [-0.34,-0.01]). Adherence scores for other DHD-CVD components were not associated with kidney function decline (Table 4). When examining absolute intakes (instead of adherence scores) of individual DHD-CVD components, nut and dairy were not significantly associated with kidney function decline, while legumes and tea consumption showed adverse associations. Reducing the intake of red meat was associated with less kidney function decline (beta of 0.20 [0.03, 0.38] per 1-SD of 23.2 g/d), in line with the results for the adherence score (Table 4).

**Table 4:** The association between components of the DHD-CVD index^a^ and differences in annual eGFR change in 2169 patients of the Alpha Omega Cohort

	SD	β (95% CI)
**Vegetables**		
Per 1-SD increment in adherence score^b^	1.95 points	0.02 (-0.15,0.20)
Per 1-SD increment in intake	42.9 g/d	0.003 (-0.17,0.18)
**Fruit**		
Per 1-SD increment in adherence score^b^	3.59 points	0.08 (-0.10,0.25)
Per 1-SD increment in intake	155 g/d	0.12 (-0.06,0.30)
**Grains**		
Per 1-SD increment in adherence score^b^	2.54 points	0.02 (-0.15,0.18)
Per 1-SD decrease in refined grains intake	36.7 g/d	-0.04 (-0.26,0.18)
Per 1-SD increment in whole grains intake	59.5 g/d	-0.006 (-0.27,0.25)
**Legumes**		
Per 1-SD increment in adherence score^b^	3.67 points	-0.20 (-0.37,-0.04)
Consumers (*n* = 1135) vs. non-consumers (*n* = 1034)	NA	-0.57 (-0.90,-0.25)
**Nuts**		
Per 1-SD increment in adherence score^b^	2.52 points	0.17 (-0.004,0.34)
Consumers (*n* = 1507) vs. non-consumers (*n* = 662)	NA	0.09 (-0.27,0.46)
**Dairy**		
Per 1-SD increment in adherence score^b^	3.06 points	-0.18 (-0.34,-0.01)
Per 1-SD increment in intake	242 g/d	-0.12 (-0.32,0.07)
**Fish**		
Per 1-SD increment in adherence score^b^	3.57 points	-0.07 (-0.24,0.10)
Per 1-SD increment in intake	15.2 g/d	-0.12 (-0.29,0.05)
Consumers (*n* = 1764) vs. non-consumers (*n* = 405)	NA	-0.20 (-0.62,0.22)
**Tea**		
Per 1-SD increment in adherence score^b^	4.06 points	-0.11 (-0.28,0.06)
Per 1-SD increment in intake	258 g/d	-0.20 (-0.37,-0.03)
**Fats and oils**		
Per 1-SD increment in adherence score^b^	4.41 points	-0.10 (-0.27,0.07)
Per 1-SD increment in liquid fat intake	17.6 g/d	0.13 (-0.09,0.35)
Per 1-SD decrease in solid fat intake	18.3 g/d	-0.17 (-0.38,0.05)
**Red meat**		
Per 1-SD increment in adherence score^b^	2.01 points	0.21 (0.04,0.38)
Per 1-SD decrease in intake	23.2 g/d	0.20 (0.03,0.38)
**Processed meat**		
Per 1-SD increment in adherence score^b^	3.25 points	0.01 (-0.18,0.20)
Per 1-SD decrease in intake	21.4 g/d	-0.04 (-0.25,0.17)
**Sugar-sweetened beverages and fruit juices**		
Per 1-SD increment in adherence score^b^	3.35 points	-0.05 (-0.22,0.12)
Per 1-SD decrease in intake	211 g/d	-0.13 (-0.30,0.06)
**Alcohol**		
Per 1-SD increment in adherence score^b^	3.90 points	0.11 (-0.07,0.29)
Per 1-SD decrease in intake	15.6 g/d	0.06 (-0.14,0.26)
**Sodium**		
Per 1-SD increment in adherence score^b^	2.68 points	-0.06 (-0.30,0.19)
Per 1-SD decrease in intake	659 mg/d	-0.004 (-0.36,0.35)
**Plant sterols or stanol-enriched products**		
Per 1-SD increment in intake	14.1 g/d	-0.08 (-0.42,0.26)
Consumers (*n* = 877) vs. non-consumers (*n* = 1292)	NA	-0.05 (-0.23,0.13)

^a^ Classification of foods and drinks included in the DHD-CVD index is listed in Supplemental Table 3. ^b^ A higher score means better adherence to the dietary guideline for that specific component. Abbreviations: DHD-CVD, Dutch Healthy Diet for cardiovascular disease patients; eGFR, estimated glomerular filtration rate; SD, standard deviation; NA, not applicable

### Diet quality and kidney function decline in strata of genetic CKD risk

The distributions of genetic risk scores (i.e. GRS_all based on 88 SNPs and GRS_sub based on 16 SNPs) are shown in Supplemental Fig. 3. The total DHD-CVD score was generally similar across strata of GRS_all and GRS_sub (Supplemental Table 5, Supplemental Table 6).

The overall DHD-CVD index was not associated with kidney function decline in strata of genetic CKD risk (Table 5). In patients with a high genetic CKD risk, associations for adherence to guidelines for legumes, nut, and dairy were no longer present. Adherence to the guideline for reducing red meat intake was associated with less kidney function decline according to GRS_all (beta of 0.31 [0.00,0.61] per 1-SD, *n* = 709), but no association was observed according to GRS_sub (beta of 0.15 [-0.09,0.39] per 1-SD, *n* = 1063) (Supplemental Table 7). When examining absolute intakes (instead of adherence scores) of individual components among patients with a high genetic CKD risk, intake of legumes, dairy, and alcohol were not associated with kidney function decline. Results for nut consumption and reduction of red meat intake suggested (a trend towards) less kidney function decline according to GRS_all (beta_nut consumers vs. non−consumers_ of 0.53 [-0.12,1.19] and beta_red meat reduction per 23.0 g/d_ of 0.33 [0.01,0.65]), but no association was observed according to GRS_sub (beta_nut consumers vs. non−consumers_ of 0.08 [-0.44,0.61] and beta_red meat reduction per 23.3 g/d_ of 0.15 [-0.11,0.41]) (Supplemental Table 7).

**Table 5:** The association between the DHD-CVD index per 1-SD increment in adherence score and differences in annual eGFR change in patients of the Alpha Omega Cohort, stratified by categories of genetic risk of CKD

	Beta per 1-SD increment in DHD-CVD adherence score	
	GRS_all^a^	GRS_sub^d^
**Low genetic risk of CKD**		
Range	≥-4.161-≤-0.434	≥-3.425-≤-0.00105
Sample size	*N* = 709	*N* = 1063
Mean ± SD annual eGFR change	-1.87 ± 3.98	-1.73 ± 3.76
Multivariable model^b^	0.05 (-0.26,0.36)^c^	0.003 (-0.24,0.25)
**Intermediate genetic risk of CKD**		
Range	>-0.434–≤0.411	NA
Sample size	*N* = 708	NA
Mean ± SD annual eGFR change	-1.61 ± 3.64	NA
Multivariable model^b^	-0.07 (-0.35,0.21)	NA
**High genetic risk of CKD**		
Range	> 0.411 – ≤3.950	>-0.00105– ≤3.572
Sample size	*N* = 709	*N* = 1063
Mean ± SD annual eGFR change	-1.69 ± 3.91	-1.72 ± 3.93
Multivariable model^b^	-0.15 (-0.47,0.16)	-0.16 (-0.41,0.09)

^a^ GRS_all is defined as a genetic risk score based on 88 non-ambiguous SNPs that are both nominally and genome-wide significantly associated with CKD. ^b^ Adjusted for age, sex, education, energy intake, smoking status, physical activity, lipid-lowering medication use, renin-angiotensin-aldosterone system blockers, and the first three genetic principal components. ^c^ Beta coefficient (95% confidence interval) obtained from linear regression models (all such values). ^d^ GRS_sub is defined as a genetic risk score based on 16 non-ambiguous SNPs that are genome-wide significantly associated with CKD. Abbreviations: DHD-CVD index, Dutch Healthy Diet for cardiovascular disease patients; SD, standard deviation; eGFR, estimated glomerular filtration rate; CKD, chronic kidney disease

### Additional analyses

For the total DHD-CVD index, results remained generally similar in subgroups of patients with diabetes, obesity, or CKD (Supplemental Table 8) and also after excluding users of RAAS inhibitors or (loop) diuretics (Supplemental Table 9). For individual DHD-CVD components, results varied somewhat in several subgroups of patients with diabetes, obesity, or CKD (Supplemental Tables 10–12). Most associations in these subgroups were not statistically significant. However, associations between legumes (higher scores and intake), tea (higher intake), and alcohol (higher scores) and kidney function decline tended to be more pronounced among 484 patients with obesity (Supplemental Table 11) as compared to the total cohort. The association between dairy (higher intake) and kidney function decline was more pronounced in 273 patients with prevalent CKD (Supplemental Table 12).

## Discussion

In this prospective cohort study of drug-treated post-MI patients, overall adherence to dietary guidelines, adapted for CVD patients, was not significantly associated with kidney function decline. Generally similar results were found across strata of genetic CKD risk. Of the 15 specific DHD-CVD components that were examined in this study, less kidney function decline was observed when patients adhered to guidelines for higher nut consumption and lower red meat intake. More kidney function decline was found in patients who adhered to guidelines for legumes and dairy.

Our main result for overall adherence to dietary guidelines and kidney function is in line with two population-based studies that examined diet quality scores in relation to kidney function outcomes [27, 28]. A healthy diet score that was part of the American Heart Association’s Life’s Simple 7 was not associated with incident CKD after 22 years of follow-up in US black and white women [27]. The Alternate Healthy Eating Index 2010, Dietary Approaches to Stop Hypertension, and the Mediterranean diet scores were also not associated with incident CKD after six years of follow-up among US Hispanics and Latinos [28]. In contrast to our findings, results of the CORDIOPREV trial among coronary heart disease patients showed that a Mediterranean diet supplemented with extra-virgin olive oil was more effective in reducing eGFR decline compared to a low-fat diet rich in complex carbohydrates after five years of follow-up [6]. Patients assigned to the Mediterranean diet were recommended to consume ≥ 450 g/d of fruit and ≥ 400 g/d of vegetables [29]. In our dietary score, the DHD-CVD index, a maximum score was assigned to those consuming ≥ 200 g/d of fruit and ≥ 200 g/d of vegetables. Therefore, it is possible that higher intakes of vegetables and fruit are needed to exert beneficial effects on kidney function in cardiovascular patients. Another explanation for our null findings could be related to the consumption of tea or dairy, for which we found a potential adverse association in the current analysis. In the Mediterranean diet of the CORDIOPREV trial, tea and dairy components were not included [29].

Adherence to guidelines for limiting red meat consumption was associated with less kidney function decline. Red meat contains animal protein, which has been associated with accelerated kidney function decline in a previous Alpha Omega Cohort analysis [30]. Red meat intake was also associated with a higher incidence of CKD and kidney failure in the population-based Atherosclerosis Risk In Communities (ARIC) Study (median intake: ~0.60 US servings/day; 22 years of follow-up) [31] and the Singapore Chinese Health Study (median intake: ~30 g/d; 15.5 years of follow-up) [32]. In our cohort, included foods were steak, pork fillet, and minced meat, and intakes were low (~ 37 g/d). Although studies in patients with CVD are lacking for comparison, our findings may highlight the importance of limiting red meat intake for slowing down kidney function decline after MI.

Better adherence to the guideline for nut intake (≥ 15 g/d) was associated with less kidney function decline in our study. Previous population-based studies also showed potential health benefits of nut consumption on kidney function in US populations [31, 33]. Underlying mechanisms could be related to less inflammation, improved insulin sensitivity, improved blood lipid profile, and vascular function [34]. Nuts are also rich in fiber and unsaturated fatty acids (e.g. alpha linolenic acid), which have been associated with improved cardiometabolic health outcomes in previous studies [34]. Our cohort of patients with a history of MI consumed nuts with the main meals or as a savoury snack, including salted and unsalted peanuts, cocktail nuts, cashew nuts, walnuts, and sunflower seeds. The intake of nuts in our cohort was low, only ~ 6 g/d, whereas the median consumption of nuts in the ARIC study was about twice as high [31]. To the best of our knowledge, similar studies in patients with CVD are lacking. Our findings suggest that adherence to nut consumption guidelines could be important for slowing down kidney function decline after MI, but caution is needed when interpreting the results because we could not adjust for salt.

In our cohort, we found an unexpected adverse association for legumes in relation to kidney function decline. Legumes are considered part of a healthy diet, and their consumption is promoted in dietary guidelines. In the ARIC study, legumes were studied in relation to incident CKD [31], showing a beneficial association for the top vs. bottom quintile of intake. However, an opposite trend was found in splines analysis of the ARIC study [31]. In the Singapore Chinese Health Study, the combined intake of legumes and soy was non-significantly associated with a lower risk of kidney failure [32]. Studies of legume intake and CKD risk in patients with CVD are lacking. In our study, the intake of legumes among consumers was very low (< 10 g/d), comprising primarily of canned beans and capuchins, where salt may have been added. In the ARIC study, legumes included fresh, frozen, or canned peas or lima beans and lentils, and the median intake was 0.29 US servings/day [31], which is 3–4 times higher than in our cohort. More research into the type and amount of legume intake in relation to kidney function in CVD patient cohorts is warranted.

In the present analysis, higher dairy intake (g/d) was adversely associated with kidney function decline, particularly in patients with CKD at baseline. The DHD-CVD index (in line with the Dutch dietary guidelines) does not distinguish between low-fat and full-fat dairy products, or give recommendations for specific dairy products. In a previous analysis in the Alpha Omega Cohort, we found adverse associations for yoghurt (irrespective of fat content) with kidney function decline [35]. Our findings in patients with a history of MI stand in contrast with findings in general populations, where beneficial associations of dairy with kidney function have been found [2, 36]. There are several potential explanations for this discrepancy. Dairy is high in protein, which has been associated with CKD progression and glomerular hyperfiltration in patients with CKD [37]. Dairy is also a significant source of phosphorus. In individuals with kidney impairment, high phosphorus intake may result in hyperphosphatemia, which can have detrimental effects on kidney function, particularly in patients using phosphate-binding medication [38].

Absolute intake of black or green tea was adversely associated with kidney function decline in our cohort of post-MI patients. This adverse association was even more pronounced in patients with obesity. Similar adverse associations were observed in a previous analysis of adults with metabolic syndrome of the PREDIMED-Plus study [39]. In our study, mainly (caffeinated) black tea was consumed. Black tea has a high concentration of soluble oxalates, about 5 mg/g of tea [40]. After binding to calcium, oxalates may form crystals that turn into kidney stones [40, 41]. To what extent dietary oxalate could impact CKD risk is unclear. More research is needed to conclude whether tea could adversely impact kidney function in patients with CVD and obesity.

To our knowledge, this is the first observational study on diet quality and kidney function among CVD patients that also include data on genetic predisposition, and in which a diet quality score was applied specifically developed for CVD patients [10]. Other strengths include a relatively large cohort of patients with stable CVD, with detailed data on potential confounders, and the use of an extensive, validated FFQ. Caution is warranted in interpreting the results of sensitivity and subgroup analyses, because chance findings may be present given the large number of tests. Finally, high salt intake is an established risk factor for hypertension and kidney function decline [42, 43], but our FFQ was not a suitable instrument for salt intake because discretionary salt use could not be measured. Further, salt content varies highly across brands of processed foods for which intake could not be accurately assessed. Multiple 24-hour urine samples are needed for accurate assessment of sodium intake [44], but these were not collected in the Alpha Omega Cohort.

In conclusion, overall adherence to dietary guidelines for CVD patients showed little association with kidney function decline in Dutch CVD patients, irrespective of genetic CKD risk. We found unexpected associations for several dietary components, which need confirmation in other CVD cohorts and intervention studies, and for which potential underlying mechanisms need to be explored. More research is necessary to identify diets that support long-term health in CVD patients, without compromising kidney function.

### Electronic Supplementary Material

Below is the link to the electronic supplementary material.

Supplementary Material 1
