# Supplementary material for: Diet quality in relation to kidney function and its potential interaction with genetic risk of kidney disease among Dutch post-myocardial infarction patients
Source: Eur J Nutr. 2024 Mar 2;63(4):1373–85. doi: 10.1007/s00394-024-03355-5 (PMC11139691; doi:10.1007/s00394-024-03355-5)
Supplement: Supplementary file 1 — Supplementary Material 1 [file 394_2024_3355_MOESM1_ESM.docx]

Contents Supplementary Information:

| Supplemental Table 1 | Baseline characteristics of patients of the Alpha Omega Cohort who were included or excluded from the analysis of DVD-CVD index and kidney function decline. |
| --- | --- |
| Supplemental Table 2 | Components and Dutch dietary guidelines of the Dutch Healthy Diet Cardiovascular Disease index (DHD-CVD index) and their threshold (minimum score) and cut-off (maximum score) values. |
| Supplemental Table 3 | Classification of foods and drinks included in the DHD-CVD index in the Alpha Omega Cohort. |
| Supplemental Table 4 | List of SNPs obtained from the GWAS on kidney function, that were used to calculate a genetic risk score for 2126 patients of the Alpha Omega Cohort. |
| Supplemental Table 5 | Baseline characteristics of 2126 patients of the Alpha Omega Cohort across tertiles of the genetic risk score for CKD. |
| Supplemental Table 6 | Baseline characteristics of 2126 patients of the Alpha Omega Cohort across groups of GRS_sub for CKD. |
| Supplemental Table 7 | The association between components of the DHD-CVD index and differences in annual eGFR change in Alpha Omega Cohort patients with a high genetic risk of CKD. |
| Supplemental Table 8 | The association between the DHD-CVD index per 1-SD increment in adherence score and in sex-specific tertiles and differences in annual eGFR change in patients of the Alpha Omega Cohort, stratified by diabetes, obesity, and CKD status. |
| Supplemental Table 9 | The association between the DHD-CVD index per 1-SD increment in adherence score and in sex-specific tertiles and differences in annual eGFR change in patients of the Alpha Omega Cohort who do not use RAAS or (loop)-diuretics medication. |
| Supplemental Table 10 | The association between components of the DHD-CVD index^a^ and differences in annual eGFR change in 394 patients of the Alpha Omega Cohort with diabetes. |
| Supplemental Table 11 | The association between components of the DHD-CVD index^a^ and annual eGFR change in 484 patients of the Alpha Omega Cohort with obesity. |
| Supplemental Table 12 | The association between components of the DHD-CVD index and annual eGFR change in 273 patients of the Alpha Omega Cohort with CKD. |
| Supplemental Figure 1 | Flow chart for selection of the analytical sample of the Alpha Omega Cohort. |
| Supplemental Figure 2 | Flow chart for selection process of SNPs included in the GRS. |
| Supplemental Figure 3 | Distributions of GRS for CKD among 2126 patients of the Alpha Omega Cohort. |

| **Supplemental Table 1** Baseline characteristics of patients of the Alpha Omega Cohort who were included or excluded from the analysis of DVD-CVD index and kidney function decline. | | |
| --- | --- | --- |
|  | Included patients  (n=2169) | Excluded patients (n=2668) |
| **Sociodemographic factors** |  |  |
| Age, y | 68.9 ± 5.40 | 69.2 ± 5.70 |
| Women, n(%) | 417 (19.2) | 637 (23.9) |
| Education, n(%) |  |  |
| Only elementary | 446 (20.7) | 557 (21.1) |
| Low | 779 (36.1) | 933 (35.3) |
| Intermediate | 671 (31.1) | 821 (31.0) |
| High | 263 (12.2) | 334 (12.6) |
| **Lifestyle** |  |  |
| Smoking status, n(%) |  |  |
| Never | 360 (16.6) | 458 (17.2) |
| Former | 1481 (68.3) | 1725 (64.7) |
| Current | 328 (15.1) | 484 (18.1) |
| Physical activity, n(%) |  |  |
| Low | 856 (39.6) | 1167 (44.1) |
| Intermediate | 807 (37.4) | 965 (36.4) |
| High | 497 (23.0) | 516 (19.5) |
| **Blood lipids, mmol/L** |  |  |
| Total serum cholesterol | 4.75 [4.19, 5.33] | 4.53 [3.95, 5.23] |
| LDL-cholesterol | 2.64 [2.17, 3.17] | 2.37 [1.89, 2.94] |
| HDL-cholesterol | 1.21 [1.03, 1.43] | 1.26 [1.06, 1.50] |
| Triglycerides | 1.63 [1.21, 2.26] | 1.67 [1.22, 2.36] |
| **Other cardiovascular factors** |  |  |
| SBP, mmHg | 143 ± 21.2 | 140 ± 21.9 |
| DBP, mmHg | 81.5 ± 10.7 | 79.0 ± 11.4 |
| BMI, kg/m^2^ | 27.6 ± 3.61 | 27.9 ± 4.04 |
| Obesity, n(%) | 483 (22.3) | 687 (25.8) |
| Plasma glucose, mmol/L | 5.46 [4.96, 6.35] | 5.77 [5.18, 6.83] |
| Diabetes mellitus, n(%) | 394 (18.2) | 620 (23.2) |
| **Kidney function** |  |  |
| eGFR, mL/min per 1.73 m^2^ | 87.0 [71.4, 99.5] | 80.0 [63.5, 95.2] |
| CKD^d^, n(%) | 273 (12.6) | 488 (20.4) |
| Serum creatinine, µmol/L | 84.0 [72.0, 101.0] | 90.0 [75.0, 113.0] |
| Serum cystatin C, mg/L | 0.92 [0.82, 1.10] | 0.95 [0.84, 1.10] |
| **Medication use, n(%)** |  |  |
| Antihypertensives | 1887 (87.0) | 2453 (91.9) |
| ACE-inhibitors | 918 (42.3) | 1148 (43.0) |
| ARBs | 287 (13.2) | 427 (16.0) |
| Diuretics | 442 (20.4) | 742 (27.8) |
| Lipid-lowering agents | 1872 (86.3) | 2289 (85.8) |

Values are means ± SDs for normally distributed variables, medians [IQRs] for skewed variables, or n (%) for categorical variables. Abbreviations: LDL-cholesterol, low-density lipoprotein cholesterol; HDL-cholesterol, high-density lipoprotein cholesterol; SBP, systolic blood pressure; DBP, diastolic blood pressure; BMI, body mass index; eGFR, estimated glomerular filtration rate; CKD, chronic kidney disease; ACE-inhibitors, Angiotensin-Converting Enzyme inhibitors; ARBs, Angiotensin receptor blockers.

**Supplemental Table 2** Components and Dutch dietary guidelines of the Dutch Healthy Diet Cardiovascular Disease index (DHD-CVD index) and their threshold (minimum score) and cut-off (maximum score) values^a^.

| Component | Dutch dietary guidelines for CVD patients | Minimum score (=0 points) | Maximum score (=10 points) |
| --- | --- | --- | --- |
| Vegetables | Eat ≥200 g/d of vegetables | 0 g/d | ≥200 g/d |
| Fruit | Eat ≥200 g/d of fruit | 0 g/d | ≥200 g/d |
| Whole grain products^b^ | a. Eat ≥90 g/d of whole grain products  b. Replace refined cereal products by whole grain products | No consumption of whole grain products  OR  Ratio of whole grains to refined ≤0.7 | No consumption of refined cereal products  OR  Ratio of whole grains to refined grains ≥11 |
| Legumes | Eat legumes weekly | 0 g/d | ≥10 g/d |
| Nuts | Eat ≥15 g/d of unsalted nuts | 0 g/d | ≥15 g/d |
| Dairy^c^ | Eat a few portions of dairy products daily, including milk or yogurt | 0 g/d OR ≥750 g/d | 300-450 g/d |
| Fish | Eat 1-2 servings of fish (any type) weekly | 0 g/d | ≥21 g/d |
| Tea | Drink 3 cups of black or green tea daily | 0 g/d | ≥450 g/d |
| Fats and oils | Replace butter, hard margarines and cooking fats by soft margarines, liquid cooking fats and vegetable oils | No consumption of soft margarines, liquid cooking fats and vegetable oils  OR  Ratio of liquid cooking fats to solid cooking fats ≤0.6 | No consumption of butter, hard margarines and cooking fats  OR  Ratio of liquid cooking fats to solid cooking fats ≥13 |
| Red meat | Limit consumption of red meat | ≥100 g/d | ≤45 g/d |
| Processed meat | Limit consumption of processed meat | ≥50 g/d | 0 g/d |
| Sugar-sweetened beverages and fruit juices | Limit consumption of sugar-sweetened beverages and fruit juices | ≥250 g/d | 0 g/d |
| Alcohol | If alcohol is consumed at all, intake should be limited to one Dutch unit (10 g/d ethanol) | Women: ≥20 g/d ethanol  Men: ≥30 g/d ethanol | Women and men: ≤10 g/d ethanol |
| Salt | Limit consumption of table salt to 6 g/d | ≥3.8 g/d sodium | ≤1.9 g/d sodium |
| Plant sterol or stanol-enriched products | Consider the use of cholesterol-lowering plant sterol or stanol-enriched products | 0 g/d | >0 g/d |

^a^ Modified from Looman et al. 2017 [1]. ^b^ This component comprises two sub-components (a and b). Each sub-component has a maximum score of 5 points.

^c^ Maximum of 40 g cheese can be included.

**Supplemental Table 3** Classification of foods and drinks included in the DHD-CVD index in the Alpha Omega Cohort.

| **DHD-CVD index components** | **Food items included** |
| --- | --- |
| Vegetables | Endive, spinach and purslane, sprouts, cauliflower, broccoli, other cabbages, carrots, peas, broad beans, all sorts of green beans and string beans, leek, chicory, swede, beets, mushrooms, bell pepper, tomatoes, onion, lettuce and raw vegetables, other sorts of vegetables |
| Fruits | Citrus fruits, apples, pears, bananas, strawberries, blueberries, redcurrant, blackberries, raspberries, cherries, grapes, peaches, nectarines, plums, apricot, kiwi, pineapple, fresh cranberries, melon, mango, papaya, persimmon fruit, passion fruit, lychee, watermelon |
| Whole grains^a^ | Wheat bread, whole wheat bread, malt bread, dark and light rye bread, whole grain bread, whole wheat rye bread, whole raisin bread, whole meal rusks, fiber-rich crispbread, whole crispbread, muesli crispbread, whole wheat cracottes, regular muesli, oatmeal, breakfast product Molenaar, whole wheat macaroni, brown rice |
| Refined grains | Wasa crispbread, breakfast product All Bran Kellogg’s, Brinta, Cornflakes Kellogg’s, Rice Krispies Kellogg’s, sweetened muesli, raisin bread, white bread, Turkish white bread, croissants, grinded rice, white rice, millet, parboiled rice, wheat bulgur, cooked macaroni, wheat flour |
| Legumes | (Cooked) capuchins, white beans in tomato sauce, brown beans, dried peas split pesi, cooked white/brown beans, lentils, soybeans, boiled green peas, chickpeas |
| Nuts | Nuts and seeds with and without the warm meal, i.e. almonds, cashew nuts, hazelnuts, brazil nuts, unsalted peanuts, walnuts, unsalted mixed nuts, sunflower seeds, salted peanuts, nuts |
| Dairy | Full-fat luxury cheese, reduced fat luxury cheese,  cheese with the warm meal, cubes of cheese,  20% fat cheese, 30% fat cheese, regular cheese,  other cheese, packaged breakfast yogurt, low-fat/semi-skimmed/full-fat yogurt, custard, other types of yogurt, low-fat/semi-skimmed/full-fat milk, other types of milk, regular low-fat/semi-skimmed/full-fat milk in coffee, buttermilk, low-fat/semi-skimmed/full-fat chocolate milk and yogurt drink, other chocolate milk and yogurt drink, pudding, porridge, ice cream and other ice cream, whipped cream, coffee creamer, low-fat/semi-skimmed coffee milk, evaporated milk/coffee milk powder, unknown coffee milk, other coffee milk, cream with the warm meal |
| Fish | Fish fingers, plaice, cod, grilled whole herring, codfish, low-fat/semi-skimmed/fatty fish, pollock, tuna, sole, anchovy, trout, pan herring, buckling, canned herring fillet in tomato sauce, salted herring, eel, sardines, salmon, mackerel, herring in sour, halibut |
| Tea | Green and black tea |
| Fats and oils | Liquid cooking fats and oils  Different types of Halvarine products, different types of margarine products, Halvarine and margarine products enriched with plant sterols/stanols, tub margarine 70% fat >17 g saturated fatty acids, diet margarine 60 and 70% fat <17 g saturated fatty acids, sunflower oil, corn oil, soybean oil, safflower oil, peanut oil, olive oil, liquid frying fat, tub margarine 70% fat >17 g saturated fatty acids, liquid bake and frying fats 97% fat, liquid margarine 80% fat <17 g saturated fatty acids,  Solid cooking fats  Tub margarine 80% fat 17-24 g saturated fatty acids, salted and unsalted butter, semi-skimmed butter, stick margarine 80% fat >24 g saturated fatty acids, solid frying fat, solid bake and frying fats, bacon fat |
| Sugar-sweetened beverages and fruit juices | Alcohol-free beer, orange juice, apple juice, grape juice, grapefruit juice, tomato juice, vegetable juice, other juices, rosehip syrup, coke with caffeine, other soda’s, sport drinks, breakfast drinks with fruits, low-fat/semi-skimmed/full-fat chocolate milk, milkshake, yogurt drinks, buttermilk with fruits |
| Unprocessed red meat | Different types of organ meat, steaks, pork meat, pork fillet, pork ribs, minced meat, sheep |
| Processed red meat | Prepared organ meat (liver), beef (loin) roulade, blind veal finch, salted beef, pork fricandeau, pork ham slice, cooked liver, liver products, gammon, luncheon meat, bacon, sausage |
| Alcohol | Pie or cake, beer, low alcohol or alcohol-free beer, advocaat, mixed/longdrinks, strong liquor,  pudding, pieces of chocolates, red wine, rosé wine, white wine, sherry, vermouth, port |
| Sodium^b^ | Cooked liver, bacon, unknown types of meat, sausage, mustard |
| Plant sterol or stanol-enriched products | Halvarine Becel pro.activ, margarine Benecol, Halvarine Benecol light |

^a^ Foods were categorized as whole grain product if they contained at least 25 % wholegrain flour.

^b^ Sodium intake was only estimated from foods, because discretionary salt use could not be assessed by means of the FFQ. Sodium is present in all food items of the FFQ, except for other types of oil, solid deep frying oil, liquid deep frying oil, lard, olive oil, and deep frying oil. The food items mentioned in the table, are the foods that contribute the most to total sodium intake. Abbreviations: DHD-CVD, Dutch Healthy Diet for cardiovascular disease patients.

**Supplemental Table 4** List of SNPs obtained from the GWAS on kidney function^a^, that were used to calculate a genetic risk score for 2126 patients of the Alpha Omega Cohort.

| **SNPs obtained from GWAS** | **SNPs available in the**  **Alpha Omega Cohort and not ambiguous (GRS_all)** | **Genome-wide significant and not ambiguous (GRS_sub)^b^** |
| --- | --- | --- |
| rs74748843 | rs74748843 |  |
| rs17413465 | rs17413465 |  |
| rs1757915 | rs1757915 |  |
| rs679843 | rs679843 |  |
| rs11166440 | rs11166440 |  |
| rs267738 | rs267738 |  |
| rs4971100 | rs4971100 |  |
| rs3850625 | rs3850625 |  |
| rs2808454 |  |  |
| rs2490391 | rs2490391 | Yes |
| rs3791221 | rs3791221 |  |
| rs807624 | rs807624 |  |
| rs6546869 | rs6546869 | Yes |
| rs11123169 | rs11123169 | Yes |
| rs11694902 | rs11694902 |  |
| rs7425436 | rs7425436 |  |
| rs35472707 | rs35472707 |  |
| rs187355703 |  |  |
| rs35284526 | rs35284526 |  |
| rs4666821 | rs4666821 |  |
| rs7651407 | rs7651407 |  |
| rs3774726 | rs3774726 |  |
| rs2289746 | rs2289746 |  |
| rs9868185 | rs9868185 |  |
| rs56065557 |  |  |
| rs11919484 | rs11919484 |  |
| rs9823161 | rs9823161 |  |
| rs16874073 | rs16874073 |  |
| rs28817415 | rs28817415 | Yes |
| rs12509595 | rs12509595 | Yes |
| rs223471 |  |  |
| rs13157326 | rs13157326 |  |
| rs1362800 | rs1362800 | Yes |
| rs11746506 | rs11746506 |  |
| rs12520984 |  |  |
| rs79760705 | rs79760705 |  |
| rs72759880 | rs72759880 |  |
| rs2010352 | rs2010352 |  |
| rs12163971 | rs12163971 |  |
| rs11743174 | rs11743174 |  |
| rs3812036 | rs3812036 | Yes |
| rs3765502 |  |  |
| rs144100226 |  |  |
| rs13200335 |  |  |
| rs77915916 |  |  |
| rs720989 |  |  |
| rs6458868 |  |  |
| rs3925003 |  |  |
| rs72912510 |  |  |
| rs1857859 |  |  |
| rs7740107 |  |  |
| rs3822939 |  |  |
| rs62435145 | rs62435145 | Yes |
| rs6968554 | rs6968554 |  |
| rs700753 |  |  |
| rs55773927 | rs55773927 |  |
| rs801193 | rs801193 |  |
| rs41301394 | rs41301394 |  |
| rs6973656 | rs6973656 |  |
| rs62491533 | rs62491533 |  |
| rs10254101 | rs10254101 | Yes |
| rs34861762 | rs34861762 |  |
| rs10102889 |  |  |
| rs2039424 | rs2039424 | Yes |
| rs1321917 |  |  |
| rs7024579 | rs7024579 |  |
| rs80282103 |  |  |
| rs7072591 | rs7072591 |  |
| rs10821905 | rs10821905 |  |
| rs10821944 | rs10821944 |  |
| rs7475348 | rs7475348 |  |
| rs12240572 |  |  |
| rs7095954 |  |  |
| rs2068888 | rs2068888 |  |
| rs4918943 | rs4918943 |  |
| rs284859 | rs284859 |  |
| rs1055256 | rs1055256 |  |
| rs11564722 | rs11564722 |  |
| rs963837 | rs963837 | Yes |
| rs6484504 | rs6484504 |  |
| rs61897431 | rs61897431 |  |
| rs7127946 | rs7127946 | Yes |
| rs2727040 | rs2727040 |  |
| rs1813937 | rs1813937 |  |
| rs3892895 | rs3892895 |  |
| rs11237450 | rs11237450 |  |
| rs10790452 | rs10790452 |  |
| rs632887 | rs632887 |  |
| rs117113238 | rs117113238 |  |
| rs10846157 | rs10846157 |  |
| rs12313306 | rs12313306 |  |
| rs1275609 | rs1275609 |  |
| rs690428 | rs690428 | Yes |
| rs11071738 | rs11071738 |  |
| rs351237 | rs351237 |  |
| rs4886696 |  |  |
| rs4886755 | rs4886755 |  |
| rs438339 | rs438339 |  |
| rs77924615 | rs77924615 | Yes |
| rs9932625 | rs9932625 |  |
| rs62050038 |  |  |
| rs28581385 |  |  |
| rs28735420 | rs28735420 |  |
| rs2411192 |  |  |
| rs9903801 |  |  |
| rs8866 |  |  |
| rs16942751 | rs16942751 | Yes |
| rs8096658 |  |  |
| rs7251730 | rs7251730 |  |
| rs78241494 | rs78241494 |  |
| rs113445505 | rs113445505 |  |
| rs17216707 | rs17216707 |  |
| rs2235826 |  |  |
| rs1407040 | rs1407040 |  |
| rs4408777 | rs4408777 |  |
| rs2823139 | rs2823139 | Yes |
| rs2834317 | rs2834317 |  |
| rs4820324 |  |  |
| rs738527 | rs738527 |  |

^a^ We are referring to the GWAS of Wuttke et al. 2019 [2]. ^b^ Genome-wide significant SNPs are defined as SNPs with p-value <10^-8^. Abbreviations: SNPs, single nucleotide polymorphisms; GWAS, genome-wide association study.

**Supplemental Table 5** Baseline characteristics of 2126 patients of the Alpha Omega Cohort across tertiles of the genetic risk score for CKD.

|  | GRS_all^a^ | | |
| --- | --- | --- | --- |
|  | Low risk  ≤-0.434  N=709 | Intermediate risk  >-0.434 – ≤0.411  N=708 | High risk  >0.411  N=709 |
| GRS_all | -1.10 ± 0.56 | -0.02 ± 0.24 | 1.11 ± 0.57 |
| Total DHD-CVD score | 79.7 ± 14.4 | 78.9 ± 15.1 | 79.8 ± 14.3 |
| **Sociodemographic factors** |  |  |  |
| Education^b^, n(%) |  |  |  |
| Only elementary | 133 (18.8) | 152 (21.6) | 154 (21.8) |
| Low | 266 (37.7) | 255 (36.2) | 246 (34.8) |
| Intermediate | 228 (32.3) | 217 (30.8) | 209 (29.6) |
| High | 79 (11.2) | 81 (11.5) | 97 (13.7) |
| **Lifestyle** |  |  |  |
| Smoking status, n(%) |  |  |  |
| Never | 108 (15.2) | 118 (16.7) | 125 (17.6) |
| Former | 516 (72.8) | 475 (67.1) | 463 (65.3) |
| Current | 85 (12.0) | 115 (16.2) | 121 (17.1) |
| Physical activity^b^, n(%) |  |  |  |
| Low | 277 (39.3) | 282 (40.0) | 279 (39.5) |
| Intermediate | 263 (37.3) | 267 (37.9) | 259 (36.6) |
| High | 165 (23.4) | 156 (22.1) | 169 (23.9) |
| **Clinical factors** |  |  |  |
| SBP^b^, mmHg | 144 ± 21.7 | 144 ± 19.9 | 143 ± 21.9 |
| DBP^b^, mmHg | 82.0 ± 10.6 | 81.5 ± 10.9 | 81.1 ± 10.6 |
| BMI^b^, kg/m^2^ | 27.7 ± 3.65 | 27.8 ± 3.60 | 27.4 ± 3.51 |
| Obesity^b, c^, n(%) | 161 (22.7) | 172 (24.3) | 137 (19.3) |
| Diabetes mellitus^d^, n(%) | 125 (17.6) | 135 (19.1) | 122 (17.2) |
| **Kidney function** |  |  |  |
| 2021 eGFR_cr-cysC_ mL/min per 1.73 m^2^ | 83.8 [69.8, 95.9] | 85.6 [69.6, 99.8] | 91.1 [75.9, 102.2] |
| 2021 eGFR_cr_ mL/min per 1.73 m^2^ | 79.2 [63.0, 92.7] | 81.5 [64.3, 95.2] | 87.5 [70.3, 96.1] |
| 2012 eGFR_cr_ mL/min per 1.73 m^2^ | 74.5 [64.4, 85.4] | 77.9 [64.6, 87.9] | 80.8 [69.5, 89.8] |
| Serum creatinine, µmol/L | 87.0 [75.0, 104.0] | 85.0 [71.0, 103.0] | 80.0 [68.0, 95.0] |
| Serum cystatin C, mg/L | 0.95 [0.84, 1.10] | 0.92 [0.82, 1.10] | 0.89 [0.80, 1.00] |

Values are means ± SDs for normally distributed variables, medians [IQRs] for skewed variables, or n(%) for categorical variables. ^a^ GRS_all is defined as a genetic risk score based on 88 non-ambiguous SNPs that are both nominally and genome-wide significantly associated with CKD. ^b^ Part of the cohort had missing values for education (n=9), physical activity (n=9), SBP (n=3), DBP (n=3), BMI and obesity (n=2). ^c^ Obesity is defined as BMI ≥30 kg/m^2^. ^d^ Diabetes mellitus is defined as a self-reported physician’s diagnosis, use of glucose-lowering medication or elevated plasma glucose (≥7.0 mmol/L if fasted >4 h or ≥11.0 mmol/L if not fasted). Abbreviations: GRS, genetic risk score; DHD-CVD, Dutch Healthy Diet for cardiovascular disease patients; SBP, systolic blood pressure; DBP, diastolic blood pressure, BMI, body mass index; eGFR, estimated glomerular filtration rate; CKD, chronic kidney disease.

**Supplemental Table 6** Baseline characteristics of 2126 patients of the Alpha Omega Cohort across groups of GRS_sub for CKD.

|  | GRS_sub | |
| --- | --- | --- |
|  | Low risk  ≤-0.00105  N=1063 | High risk  >-0.00105  N=1063 |
| GRS_sub^a^ | -0.80 ± 0.58 | 0.80 ± 0.64 |
| Total DHD-CVD score | 79.3 ± 14.4 | 79.6 ± 14.7 |
| **Sociodemographic factors** |  |  |
| Education^b^, n(%) |  |  |
| Only elementary | 208 (19.6) | 231 (21.9) |
| Low | 399 (37.6) | 368 (34.8) |
| Intermediate | 324 (30.6) | 330 (31.2) |
| High | 129 (12.2) | 128 (12.1) |
| **Lifestyle** |  |  |
| Smoking status, n(%) |  |  |
| Never | 166 (15.6) | 185 (17.4) |
| Former | 736 (69.2) | 718 (67.5) |
| Current | 161 (15.1) | 160 (15.1) |
| Physical activity^b^, n(%) |  |  |
| Low | 407 (38.5) | 431 (40.7) |
| Intermediate | 406 (38.4) | 383 (36.2) |
| High | 245 (23.2) | 245 (23.1) |
| **Clinical factors** |  |  |
| SBP^b^, mmHg | 143 ± 20.3 | 144 ± 22.1 |
| DBP^b^, mmHg | 81.8 ± 10.6 | 81.2 ± 10.8 |
| BMI^b^, kg/m^2^ | 27.7 ± 3.52 | 27.6 ± 3.66 |
| Obesity^b, c^, n(%) | 238 (22.4) | 232 (21.8) |
| Diabetes mellitus^d^, n(%) | 173 (16.3) | 209 (19.7) |
| **Kidney function** |  |  |
| 2021 eGFR_cr-cysC_ mL/min per 1.73 m^2^ | 85.2 [70.4, 98.4] | 88.5 [72.2, 100.8] |
| 2021 eGFR_cr_ mL/min per 1.73 m^2^ | 80.5 [63.6, 94.5] | 84.0 [68.0, 95.5] |
| 2012 eGFR_cr_ mL/min per 1.73 m^2^ | 75.8 [64.6, 86.7] | 79.4 [67.5, 88.8] |
| Serum creatinine, µmol/L | 85.0 [73.0, 103.0] | 82.0 [70.0, 98.0] |
| Serum cystatin C, mg/L | 0.93 [0.83, 1.10] | 0.90 [0.81, 1.00] |

Values are means ± SDs for normally distributed variables, medians [IQRs] for skewed variables, or n(%) for categorical variables. ^a^ GRS_sub is defined as a genetic risk score based on 16 non-ambiguous SNPs that are genome-wide significantly associated with CKD. ^b^ Part of the cohort had missing values for education (n=9), physical activity (n=9), SBP (n=3), DBP (n=3), BMI and obesity (n=2). ^c^ Obesity is defined as BMI ≥30 kg/m^2^. ^d^ Diabetes mellitus is defined as a self-reported physician’s diagnosis, use of glucose-lowering medication or elevated plasma glucose (≥7.0 mmol/L if fasted >4 h or ≥11.0 mmol/L if not fasted). Abbreviations: GRS, genetic risk score; DHD-CVD, Dutch Healthy Diet for cardiovascular disease patients; SBP, systolic blood pressure; DBP, diastolic blood pressure, BMI, body mass index; eGFR, estimated glomerular filtration rate; CKD, chronic kidney disease.

**Supplemental Table 7** The association between components of the DHD-CVD index^a^ and differences in annual eGFR change in Alpha Omega Cohort patients with a high genetic risk of CKD.

|  | GRS_all^b^ | | |  | GRS_sub^c^ | | |
| --- | --- | --- | --- | --- | --- | --- | --- |
|  | Sample size | SD | β (95% CI) |  | Sample size | SD | β (95% CI) |
| **Legumes** |  |  |  |  |  |  |  |
| Per 1-SD increment in adherence score^d^ |  | 3.69 points | 0.03 (-0.27,0.33) |  |  | 3.64 points | -0.09 (-0.34,0.16) |
| Consumers vs non-consumers | 376 vs 333 | NA | -0.06 (-0.65,0.53) |  | 551 vs 512 | NA | -0.35 (-0.83,0.13) |
|  |  |  |  |  |  |  |  |
| **Nuts** |  |  |  |  |  |  |  |
| Per 1-SD increment in adherence score^d^ |  | 2.42 points | 0.14 (-0.18,0.46) |  |  | 2.53 points | 0.15 (-0.10,0.40) |
| Consumers vs non-consumers | 486 vs 223 | NA | 0.53 (-0.12,1.19) |  | 725 vs 338 | NA | 0.08 (-0.44,0.61) |
|  |  |  |  |  |  |  |  |
| **Dairy** |  |  |  |  |  |  |  |
| Per 1-SD increment in adherence score^d^ |  | 3.08 points | -0.08 (-0.38,0.22) |  |  | 3.14 points | -0.14 (-0.38,0.10) |
| Per 1-SD increment in intake |  | 229 g/d | -0.07 (-0.41,0.27) |  |  | 230 g/d | -0.07 (-0.35,0.21) |
|  |  |  |  |  |  |  |  |
| **Tea** |  |  |  |  |  |  |  |
| Per 1-SD increment in adherence score^d^ |  | 4.09 points | -0.22 (-0.52,0.08) |  |  | 4.04 points | -0.12 (-0.37,0.13) |
| Per 1-SD increment in intake |  | 251 g/d | -0.26 (-0.57,0.06) |  |  | 256 g/d | -0.16 (-0.41,0.09) |
|  |  |  |  |  |  |  |  |
| **Red meat** |  |  |  |  |  |  |  |
| Per 1-SD increment in adherence score^d^ |  | 2.00 points | 0.31 (0.00,0.61) |  |  | 2.06 points | 0.15 (-0.09,0.39) |
| Per 1-SD decrease in intake |  | 23.0 g/d | 0.33 (0.01,0.65) |  |  | 23.3 g/d | 0.15 (-0.11,0.41) |
|  |  |  |  |  |  |  |  |
| **Alcohol** |  |  |  |  |  |  |  |
| Per 1-SD increment in adherence score^d^ |  | 4.01 points | 0.13 (-0.19,0.45) |  |  | 3.92 points | -0.02 (-0.29,0.24) |
| Per 1-SD decrease in intake |  | 15.1 g/d | 0.10 (-0.27,0.48) |  |  | 15.8 g/d | -0.13 (-0.42,0.17) |

^a^ Classification of foods and drinks included in the DHD-CVD index is listed in Supplemental Table 3. ^b^ GRS_all is defined as a genetic risk score based on 88 non-ambiguous SNPs that are both nominally and genome-wide significantly associated with CKD. Within GRS_all, high genetic risk is defined as scores ≥0.411. ^c^ GRS_sub is defined as a genetic risk score based on 16 non-ambiguous SNPs that are genome-wide significantly associated with CKD. Within GRS_sub, high genetic risk is defined as scores >-0.00105. ^d^ A higher score means better adherence to the dietary guideline for that specific component. Abbreviations: DHD-CVD index, Dutch Healthy Diet for cardiovascular disease patients; eGFR, estimated glomerular filtration rate; CKD, chronic kidney disease; SD, standard deviation.

**Supplemental Table 8** The association between the DHD-CVD index per 1-SD increment in adherence score and in sex-specific tertiles and differences in annual eGFR change in patients of the Alpha Omega Cohort, stratified by diabetes, obesity, and CKD status.

|  |  | DHD-CVD index | | |
| --- | --- | --- | --- | --- |
|  | Per 1-SD increment in adherence score | T1 | T2 | T3 |
| **Diabetes** |  |  |  |  |
| **No** |  |  |  |  |
| Sample size | N=1775 | N=589 | N=587 | N=599 |
| Mean ± SD annual eGFR change, mL/min per 1.73 m^2^ | -1.56 ± 3.75 | -1.57 ± 3.97 | -1.41 ± 3.64 | -1.69 ± 3.63 |
| Model 2^a^ | -0.10 (-0.28,0.09)^b^ | Ref | 0.22 (-0.22,0.65) | -0.08 (-0.52,0.36) |
| **Yes** |  |  |  |  |
| Sample size | N=394 | 134 | 136 | 124 |
| Mean ± SD annual eGFR change, mL/min per 1.73 m^2^ | -2.40 ± 4.24 | -2.36 ± 4.23 | -2.26 ± 4.02 | -2.60 ± 4.50 |
| Model 2^a^ | -0.09 (-0.52,0.34) | Ref | 0.21 (-0.84,1.25) | -0.18 (-1.26,0.90) |
|  |  |  |  |  |
| **Obesity** |  |  |  |  |
| **No** |  |  |  |  |
| Sample size | N=1685 | N=547 | N=558 | N=580 |
| Mean ± SD annual eGFR change mL/min per 1.73 m^2^ | -1.64 ± 3.83 | -1.58 ± 3.98 | -1.54 ± 3.77 | -1.80 ± 3.75 |
| Model 2^a^ | -0.08 (-0.27,0.11) | Ref | 0.12 (-0.33,0.58) | -0.14 (-0.60,0.58) |
| **Yes** |  |  |  |  |
| Sample size | N=484 | N=176 | N=165 | N=143 |
| Mean ± SD annual eGFR change mL/min per 1.73 m^2^ | -1.94 ± 3.93 | -2.13 ± 4.14 | -1.68 ± 3.58 | -2.01 ± 4.06 |
| Model 2^a^ | -0.07 (-0.46,0.32) | Ref | 0.39 (-0.47,1.24) | 0.12 (-0.78,1.03) |
|  |  |  |  |  |
| **CKD** |  |  |  |  |
| **No** |  |  |  |  |
| Sample size | N=1896 | N=638 | N=639 | N=619 |
| Mean ± SD annual eGFR change mL/min per 1.73 m^2^ | -1.92 ± 3.81 | -1.86 ± 4.00 | -1.79 ± 3.63 | -2.11 ± 3.80 |
| Model 2^a^ | -0.11 (-0.29,0.07) | Ref | 0.15 (-0.27,0.57) | -0.19 (-0.63,0.24) |
| **Yes** |  |  |  |  |
| Sample size | N=273 | N=85 | N=84 | N=104 |
| Mean ± SD annual eGFR change mL/min per 1.73 m^2^ | -0.27 ± 3.87 | -0.64 ± 4.12 | 0.09 ± 4.08 | -0.25 ± 3.47 |
| Model 2^a^ | 0.19 (-0.31,0.68) | Ref | 0.77 (-0.43,1.97) | 0.45 (-0.71,1.60) |

^a^ Adjusted for age, sex, education, total energy intake, smoking status, physical activity, lipid-lowering medication use and renin-angiotensin-aldosterone system blockers. ^b^ Beta coefficient (95% confidence interval) obtained from linear regression models (all such values). Abbreviations: DHD-CVD, Dutch Healthy Diet for cardiovascular disease patients; SD, standard deviation; eGFR, estimated glomerular filtration rate; CKD, chronic kidney disease.

**Supplemental Table 9** The association between the DHD-CVD index per 1-SD increment in adherence score and in sex-specific tertiles and differences in annual eGFR change in patients of the Alpha Omega Cohort who do not use RAAS or (loop)-diuretics medication.

|  |  | DHD-CVD index | | |
| --- | --- | --- | --- | --- |
|  | Per 1-SD increment in adherence score | T1 | T2 | T3 |
| **No RAAS users** |  |  |  |  |
| Sample size | N=985 | N=325 | N=323 | N=337 |
| Mean ± SD annual eGFR change mL/min per 1.73 m^2^ | -1.31 ± 3.68 | -1.26 ± 3.95 | -1.17 ± 3.57 | -1.48 ± 3.51 |
| Model 2^a^ | -0.13 (-0.38,0.11)^b^ | Ref | 0.15 (-0.43,0.73) | -0.14 (-0.74,0.45) |
|  |  |  |  |  |
| **No diuretics users** |  |  |  |  |
| Sample size | N=1727 | N=573 | N=583 | N=571 |
| Mean ± SD annual eGFR change mL/min per 1.73 m^2^ | -1.62 ± 3.61 | -1.64 ± 3.96 | -1.53 ± 3.69 | -1.70 ± 3.52 |
| Model 2^a^ | -0.08 (-0.27,0.10) | Ref | 0.13 (-0.30,0.57) | -0.07 (-0.51,0.38) |
|  |  |  |  |  |
| **No loop-diuretics users** |  |  |  |  |
| Sample size | N=1860 | N=622 | N=612 | N=626 |
| Mean ± SD annual eGFR change mL/min per 1.73 m^2^ | -1.62 ± 3.74 | -1.64 ± 3.96 | -1.50 ± 3.69 | -1.70 ± 3.55 |
| Model 2^a^ | -0.08 (-0.26,0.10) | Ref | 0.18 (-0.25,0.60) | -0.01 (-0.44,0.42) |

^a^ Adjusted for age, sex, education, total energy intake, smoking status, physical activity, lipid-lowering medication use and RAAS blockers (but not when RAAS users are excluded). ^b^ Beta coefficient (95% confidence interval) obtained from linear regression models (all such values). Abbreviations: DHD-CVD, Dutch Healthy Diet for cardiovascular disease patients; SD, standard deviation; eGFR, estimated glomerular filtration rate; RAAS, renin-angiotensin-aldosterone system blockers.

**Supplemental Table 10** The association between components of the DHD-CVD index^a^ and differences in annual eGFR change in 394 patients of the Alpha Omega Cohort with diabetes.

|  | SD | β (95% CI) |
| --- | --- | --- |
| **Legumes** |  |  |
| Per 1-SD increment in adherence score^b^ | 3.55 points | -0.28 (-0.73,0.17) |
| Consumers (n=197) vs. non-consumers (n=197) | NA | -0.65 (-1.50,0.21) |
|  |  |  |
| **Nuts** |  |  |
| Per 1-SD increment in adherence score^b^ | 2.24 points | 0.32 (-0.19,0.83) |
| Consumers (n=267) vs. non-consumers (n=127) | NA | -0.03 (-0.98,0.92) |
|  |  |  |
| **Dairy** |  |  |
| Per 1-SD increment in adherence score^b^ | 3.15 points | -0.11 (-0.54,0.32) |
| Per 1-SD increment in intake | 242 g/d | -0.02 (-0.52,0.48) |
|  |  |  |
| **Tea** |  |  |
| Per 1-SD increment in adherence score^b^ | 4.04 points | -0.35 (-0.82,0.11) |
| Per 1-SD increment in intake | 278 g/d | -0.25 (-0.66,0.17) |
|  |  |  |
| **Red meat** |  |  |
| Per 1-SD increment in adherence score^b^ | 1.98 points | 0.39 (-0.07,0.85) |
| Per 1-SD decrease in intake | 22.9 g/d | 0.24 (-0.24,0.71) |
|  |  |  |
| **Alcohol** |  |  |
| Per 1-SD increment in adherence score^b^ | 3.71 points | 0.04 (-0.46,0.55) |
| Per 1-SD decrease in intake | 16.3 g/d | -0.27 (-0.81,0.26) |

^a^ Classification of foods and drinks included in the DHD-CVD index is listed in Supplemental Table 3. ^b^ A higher score means better adherence to the dietary guideline for that specific component. Abbreviations: DHD-CVD, Dutch Healthy Diet for cardiovascular disease patients; eGFR, estimated glomerular filtration rate; MI, myocardial infarction; SD, standard deviation; NA, not applicable.

**Supplemental Table 11** The association between components of the DHD-CVD index^a^ and annual eGFR change in 484 patients of the Alpha Omega Cohort with obesity.

|  | Size of 1-SD | β (95% CI) |
| --- | --- | --- |
| **Legumes** |  |  |
| Per 1-SD increment in adherence score^b^ | 3.67 points | -0.33 (-0.70,0.05) |
| Consumers (n=260) vs. non-consumers (n=224) | NA | -0.66 (-1.38,0.06) |
|  |  |  |
| **Nuts** |  |  |
| Per 1-SD increment in adherence score^b^ | 1.93 points | 0.09 (-0.40,0.57) |
| Consumers (n=308) vs. non-consumers (n=176) | NA | -0.11 (-0.86,0.65) |
|  |  |  |
| **Dairy** |  |  |
| Per 1-SD increment in adherence score^b^ | 3.06 points | -0.04 (-0.40,0.33) |
| Per 1-SD increment in intake | 252 g/d | -0.22 (-0.63,0.18) |
|  |  |  |
| **Tea** |  |  |
| Per 1-SD increment in adherence score^b^ | 4.00 points | -0.31 (-0.69,0.07) |
| Per 1-SD increment in intake | 248 g/d | -0.43 (-0.81,-0.05) |
|  |  |  |
| **Red meat** |  |  |
| Per 1-SD increment in adherence score^b^ | 2.30 points | 0.16 (-0.17,0.49) |
| Per 1-SD decrease in intake | 24.3 g/d | -0.05 (-0.41,0.32) |
|  |  |  |
| **Alcohol** |  |  |
| Per 1-SD increment in adherence score^b^ | 3.73 points | 0.46 (0.04,0.87) |
| Per 1-SD decrease in intake | 15.0 g/d | 0.26 (-0.22,0.74) |

^a^ Classification of foods and drinks included in the DHD-CVD index is listed in Supplemental Table 3. ^b^ A higher score means better adherence to the dietary guideline for that specific component. Abbreviations: DHD-CVD, Dutch Healthy Diet for cardiovascular disease patients; eGFR, estimated glomerular filtration rate; MI, myocardial infarction; SD, standard deviation; NA, not applicable.

**Supplemental Table 12** The association between components of the DHD-CVD index^a^ and annual eGFR change in 273 patients of the Alpha Omega Cohort with CKD.

|  | Size of 1-SD | β (95% CI) |
| --- | --- | --- |
| **Legumes** |  |  |
| Per 1-SD increment in adherence score^b^ | 3.73 points | 0.02 (-0.45,0.49) |
| Consumers (n=139) vs. non-consumers (n=137) | NA | -0.44 (-1.39,0.50) |
|  |  |  |
| **Nuts** |  |  |
| Per 1-SD increment in adherence score^b^ | 2.40 points | 0.37 (-0.13,0.88) |
| Consumers (n=178) vs. non-consumers (n=98) | NA | 0.69 (-0.37,1.75) |
|  |  |  |
| **Dairy** |  |  |
| Per 1-SD increment in adherence score^b^ | 3.01 points | -0.18 (-0.67,0.30) |
| Per 1-SD increment in intake | 214 g/d | -0.68 (-1.33,-0.04) |
|  |  |  |
| **Tea** |  |  |
| Per 1-SD increment in adherence score^b^ | 4.04 points | -0.23 (-0.72,0.25) |
| Per 1-SD increment in intake | 265 g/d | -0.27 (-0.74,0.20) |
|  |  |  |
| **Red meat** |  |  |
| Per 1-SD increment in adherence score^b^ | 2.05 points | 0.32 (-0.15,0.79) |
| Per 1-SD decrease in intake | 23.8 g/d | 0.17 (-0.32,0.67) |
|  |  |  |
| **Alcohol** |  |  |
| Per 1-SD increment in adherence score^b^ | 3.14 points | 0.53 (-0.10,1.16) |
| Per 1-SD decrease in intake | 11.6 g/d | 0.63 (-0.13,1.38) |

^a^ Classification of foods and drinks included in the DHD-CVD index is listed in Supplemental Table 3. ^b^ A higher score means better adherence to the dietary guideline for that specific component. Abbreviations: DHD-CVD, Dutch Healthy Diet for cardiovascular disease patients; eGFR, estimated glomerular filtration rate; MI, myocardial infarction; SD, standard deviation; NA, not applicable.

Alpha Omega Cohort:

n=4837

Patients enrolled before August 2005*:

n=2918

Patients with two blood samples:

n=2488

No baseline blood available: n=19

No final blood available: n=178

Patients eligible for re-examination after

follow-up:

n=2685

Died during 40-months of follow-up: n=233

Analytical sample for analysis of

DHD-CVD index with kidney function decline:

n=2169

Incomplete eGFR_cr-cysC_: n=148

Incomplete dietary data: n=164

Implausible energy intake: n=7

Analytical sample for analysis of

DHD-CVD index with genetic interaction and kidney function decline:

n=2126

No genetic data available: n=43

**Supplemental Figure 1.** **Flowchart for selection of the analytical sample of the Alpha Omega Cohort.** *Due to financial constraints, only these patients were eligible for follow-up measurements. eGFR_cr-cysC_, estimated glomerular filtration rate based on creatinine and cystatin C.

SNPs obtained from GWAS:

N=119

Excluded:

N=11 unavailable SNPs in the Alpha Omega Cohort

Available SNPs in the Alpha Omega Cohort:

N=108

SNPs included in GRS_sub^b^:

N=16

Excluded:

N=20 ambiguous SNPs in the Alpha Omega Cohort

SNPs included in GRS_all^a^:

N=88

**Supplemental Figure 2.** **Flowchart for selection process of SNPs included in the GRS.** ^a^ GRS_all includes SNPs that are both nominally significantly associated (p<0.05) and genome-wide significantly associated (p<10^-8^) with CKD. ^b^ GRS_sub only includes SNPs that are genome-wide significantly associated with CKD. Abbreviations: GWAS, genome-wide association study; SNPs, single nucleotide polymorphisms; GRS, genetic risk score; CKD, chronic kidney disease.


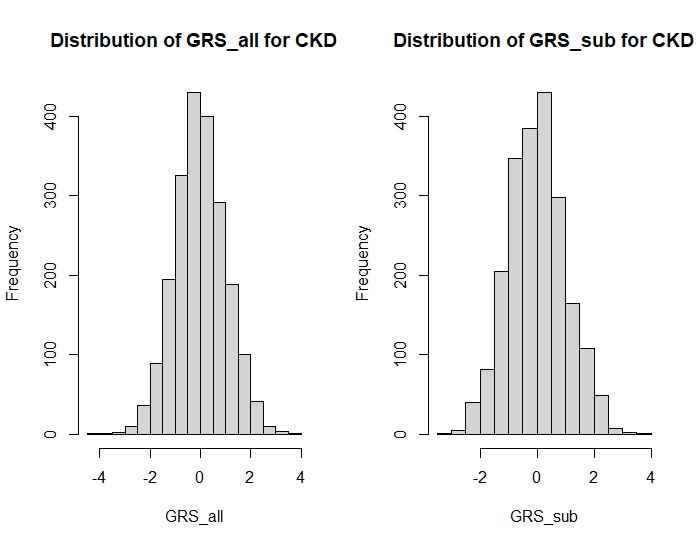


**Supplemental Figure 3. Distributions of GRS for CKD among 2126 patients of the Alpha Omega Cohort**. GRS_all ranges from -4.161 to 3.950 and GRS_sub ranges from -3.425 to 3.572. Abbreviations: GRS_all, genetic risk score based on 88 non-ambiguous SNPs that are both nominally and genome-wide significantly associated with CKD; GRS_sub, genetic risk score based on 16 non-ambiguous SNPs that are only genome-wide significantly associated with CKD; CKD, chronic kidney disease; SNP, single nucleotide polymorphism.

References

[1] Looman M, Feskens EJ, de Rijk M, Meijboom S, Biesbroek S, Temme EH et al. (2017) Development and evaluation of the Dutch Healthy Diet index 2015. Public Health Nutr 20:2289-2299. doi:10.1017/s136898001700091x

[2] Wuttke M, Li Y, Li M, Sieber KB, Feitosa MF, Gorski M et al. (2019) A catalog of genetic loci associated with kidney function from analyses of a million individuals. Nat Genet 51:957-972. doi:10.1038/s41588-019-0407-x
